# Diet

**Published:** 1899-08

**Authors:** J. A. Whitman

**Affiliations:** Charleston, S. C.


### DIET.

J. A. Whitman, M. D., Charleston, S. C.

How long would it be necessary for a physician to treat a case like the following before it could be said to be cured? The symptoms were: Bitter taste in the mouth; mouth dry, with unquenchable thirst; a condition that might be properly called "bilious." Various remedies were taken without effect.

The patient was myself. Being very fond of milk, I used it very freely in the condensed form. It occurred to me that I was taking too much of it. I then stopped its use entirely—excluding it even from my tea and coffee. The result was that in about forty-eight hours my system was restored to its normal condition. I had a similar spell two years ago. I was in the North visiting at the farm of a dairyman, where milk was as free as water. I drank much of it and ate much country cheese. In about a week it brought on the same condition. On that occasion it took four days to overcome the attack. If I had not had a knowledge of hygiene, or if ignorant of medicine I had gone to an old-school physician, he would probably have pronounced it biliousness, and I would most likely have been dosed with Calomel. How long it would have taken to relieve the condition if I had continued the use of milk is a question it would be hard to solve. As it was, stopping the cause brought about the cure of the disease. We are not always so lucky in determining the cause or in so easily bringing about the cure.

Aggravation by milk is constitutional in my case. I inherited it from my father, who for years could not take milk at all, not even in his tea and coffee.

Many of our ailments are immediately due to errors in diet. If we could determine the exact article that causes the trouble we could cure ourselves by the very simple expedient of abstaining from it. Very few persons stop to consider that what enters at the buccal cavity, sooner or later passes even through the heart, excepting, of course, what is waste material, which is rejected in the form of fecal matter. How essential is it, then, that we should keep watch and ward over what goes under our nose into the cavern below.

Thinking this experience may be an eye-opener to some other poor sufferer and set him to investigating, I give it to the readers of THE HOMŒOPATHIC PHYSICIAN for what it is worth.

